# Melatonin suppresses TLR4‐mediated RSV infection in the central nervous cells by inhibiting NLRP3 inflammasome formation and autophagy

**DOI:** 10.1111/jcmm.18338

**Published:** 2024-04-29

**Authors:** Yixuan Huang, Chengcheng Jiang, Xiaojie Liu, Wei Tang, Hongya Gui, Tao Sun, Doudou Xu, Maozhang He, Maozhen Han, Huan Qiu, Mingwei Chen, Shenghai Huang

**Affiliations:** ^1^ Department of Endocrinology The First Affiliated Hospital of Anhui Medical University Hefei China; ^2^ Department of Microbiology, School of Basic Medical Sciences Anhui Medical University Hefei China; ^3^ Department of Pediatrics The First Affiliated Hospital of Anhui Medical University Hefei China; ^4^ School of Life Sciences Anhui Medical University Hefei China; ^5^ School of Nursing Anhui Medical University Hefei China; ^6^ Department of Clinical Laboratory Anhui Public Health Clinical Center, The First Affiliated Hospital of Anhui Medical University Hefei China

**Keywords:** autophagy, inflammasome, melatonin, respiratory syncytial virus, SH‐SY5Y cells, TLR4

## Abstract

Respiratory syncytial virus (RSV) infects neuronal cells in the central nervous system (CNS), resulting in neurological symptoms. In the present study, we intended to explore the mechanism of RSV infection‐induced neuroinflammatory injury from the perspective of the immune response and sought to identify effective protective measures against the injury. The findings showed that toll‐like receptor 4 (TLR4) was activated after RSV infection in human neuronal SY5Y cells. Furthermore, TLR4 activation induced autophagy and apoptosis in neuronal cells, promoted the formation of the NOD‐like receptor family pyrin domain containing 3 (NLRP3) inflammasome, and increased the secretion of downstream inflammatory cytokines such as interleukin‐1β (IL‐1β), interleukin‐18 (IL‐18) and tumour necrosis factor‐α (TNF‐α). Interestingly, blockade of TLR4 or treatment with exogenous melatonin significantly suppressed TLR4 activation as well as TLR4‐mediated apoptosis, autophagy and immune responses. Therefore, we infer that melatonin may act on the TLR4 to ameliorate RSV‐induced neuronal injury, which provides a new therapeutic target for RSV infection.

## 1 INTRODUCTION

Respiratory syncytial virus (RSV) is an enveloped virus and is the most common pathogen causing serious lower respiratory tract infection in both infants and older adults (≥65 years).^1^, ^2^ However, RECENT advances in RSV immunobiology have not resulted in effective vaccines or clinical treatment.^3^ Clinical studies have shown that approximately 2% of RSV patients present with central nervous system (CNS) symptoms, including epilepsy, central apnea, hypersomnia, dysphagia and cerebrospinal fluid abnormalities.^4^, ^5^, ^6^, ^7^ However, the specific mechanism underlying RSV‐related encephalopathies and CNS infection has not yet been elucidated. At the same time, effective treatment options are lacking.

Toll‐like receptors (TLRs) are transmembrane signalling protein receptors that play an important role in various types of CNS inflammation.^8^ The RSV fusion protein colocalizes with TLR4 and nucleolin as demonstrated by earlier research.^9^ Simultaneously, the levels of inflammatory cytokines including IL‐6 and TNF‐α in RSV‐infected neurons were significantly increased.^9^ Additionally, the levels of inflammatory cytokines in the cerebrospinal fluid and serum of children with RSV‐related encephalopathies were markedly elevated,^10^, ^11^ suggesting that cytokines may play an important role in the occurrence of neurological complications in children with RSV. It can be concluded that RSV upregulates the expression of cytokines through the TLR4 receptor and eventually causes inflammation in the nervous system. TLR4 plays a key role in TLR/NF‐κB pathway‐induced activation of NLRP3,^12^ which prompted us to shift our research focus to NLRP3. It has been reported that TLR4 collaborated with NLRP3 to induce an efficient immune response to various stimuli. After infecting axonal neurons, RSV may enter the intracellular region via membrane fusion and undergo replication, activate host cells and mediate natural immunological reactions, causing further neuronal injury.^13^, ^14^, ^15^ It has been reported that the binding of TLR4 to the corresponding ligand eventually led to the activation of NF‐κB through a signalling pathway, which then caused the secretion of proinflammatory and chemotactic factors.^16^ TLR4 has been considered to play an important role in identifying neurodegeneration induced by synthetic single‐stranded viral RNA.^17^ In the current study, we hypothesised that microglial NLRP3 inflammasome would be activated by RSV via TLR4 and regulate neuroinflammation, a potential mechanism associated with RSV‐associated encephalopathy.

We were interested in how inflammatory cytokines might be produced during RSV‐related CNS damage, and the mechanism is still unclear. Recent studies have found that autophagy affected the inflammatory response.^11^ Saitoh et al. pointed out that the absence of the autophagy‐related protein Atg16L1 and defective autophagy resulted in the activation of Caspase‐1 and increased production of the inflammatory cytokines IL‐1β and IL‐18 by macrophages.^18^ Caspases are centrally involved in cell death and inflammatory responses.^19^ Activation of NLRP3 immediately induces the activation of Caspase‐1. Subsequently, activated Caspase‐1 cleaves the precursors of the proinflammatory cytokines IL‐1β and IL‐18, resulting in the production of their mature forms and extracellular secretion, which then triggers strong immune responses.^20^, ^21^ The autophagy‐related protein LC3, a homologue of yeast autophagy‐related gene 8 (ATG8) in mammalian cells, is targeted to the membrane of autophagic bodies and is involved in the initiation of autophagy.^22^ The LC3‐II protein has been widely used as an autophagic marker to study autophagy in mammals.^23^ The Caspase‐1/IL‐1β regulatory pathway is closely related to autophagy, which plays a crucial role in feedback regulation.^24^ This suggests that apoptosis and autophagy are important in the acceleration of inflammation.

Melatonin (MT) is a kind of indoleamine hormone synthesized and secreted by the pineal body mainly at night. MT presents beneficial effects such as anti‐free radical, anti‐oxidative stress, immunoregulatory and anti‐inflammatory effects.^25^ Studies have reported that MT regulates the NF‐кB pathway by adjusting both the activation of IкB kinase (IKK) in the early stage of formation and the binding of NF‐кB to DNA in the late stage of formation,^26^, ^27^ which may then further regulates multiple types of genes involved in the inflammatory process and the expression of cytokines.^28^ A study by Maestroni et al. suggested that MT prevents paralysis and death in mice infected with encephalomyocarditis virus after the acute stress reaction and protect the body from viral encephalitis.^29^ Semliki forest virus (SFV) is an arbovirus that causes fatal encephalitis in humans. Ben‐Nathant et al. showed that the administration of MT reduces SFV‐induced viremia, effectively delays the onset of the disease, and decreases the mortality of mice inoculated against SFV.^30^ The experiments mentioned above suggested that MT has a significant antiviral role, but whether it plays an important protective role in RSV‐related encephalopathies is still unclear.

Considering these observations collectively, we hypothesised that RSV infection of central neurons activates TLR4 and promotes NF‐кB pathway‐induced activation of NLRP3, which leads to a series of inflammatory responses that ultimately result in injuries. Additionally, we investigated whether MT alleviated the inflammatory response in central neurons by inhibiting apoptosis and autophagy and explored the important anti‐inflammatory and protective effects of MT in RSV‐related encephalopathy.

## 2 MATERIALS AND METHODS

### 2.1 Cells and virus

Human neuroblastoma cells (SH‐SY5Y cells, also called SY5Y cells) were provided by Prof. Yu‐Xian Shen (Anhui Medical University, China) and were cultured in DMEM:F12 (1:1) supplemented with 10% foetal bovine serum, 100 U/mL penicillin and 100 μg/mL streptomycin. Hep‐2 cells were provided by the Department of Microbiology of Anhui Medical University and were cultured in DMEM supplemented with 10% foetal bovine serum, penicillin and streptomycin. All cells were cultured at 37°C in a 5% CO_2_ incubator. The RSV Long strain was obtained from the Department of Microbiology of Anhui Medical University.

### 2.2 Reagents

The Annexin V Apoptosis Detection Kit was obtained from Abcam (Abcam.CN, Shanghai, China). The anti‐Caspase‐1 antibody was purchased from Bi‐Yun‐Tian Biotechnology (Haimen, China). ELISA (high sensitivity) kits for human IL‐1β, IL‐18 and TNF‐α were purchased from Neobioscience Co. (Shenzhen, China). 3‐Methyladenine (HY‐19312) (3‐MA) was purchased from MedChemExpress. MT was purchased from Sigma Chemical Co. (St. Louis, Missouri, USA).

### 2.3 Viral culture

The RSV Long strain was propagated in a monolayer of Hep‐2 cells and harvested when the cytopathic effect (CPE) was >90%. The viral titre was determined to be 2.5 × 10^−6.79^ TCID_50_/0.1 mL by the Reed‐Muench method.

### 2.4 Cell culture and virus inoculation

SY5Y cells were evenly spread at a density of 5.0 × 10^5^ cells/ml in a 12‐well cell culture plate. The RSV was inoculated when the cell sample was at approximately 80% confluence, and RSV at a TCID_50_ of 20 was uniformly adsorbed for 1 h. The previously prepared cell maintenance solution was added, and cells were cultured at 37°C in a 5% CO_2_ incubator.

### 2.5 Experimental groups

SY5Y cells were pre‐treated with TLR4 siRNA and 1 nmol/L MT, respectively. After 4, 12, 24 and 48 h of RSV infection, the protein, mRNA and cell culture supernatant of each experimental group were collected for experiments. The experiments were replicated three times. A total of four experimental groups were administered as follows: (i) normal control group, (ii) RSV‐infected group, (iii) RSV‐infected group pre‐treated with TLR4 siRNA, (iv) RSV‐infected group pre‐treated with MT.

### 2.6 Cell transfection

The TLR4 siRNA sequences are listed as follows: TLR4 siRNA‐1, 5'‐GCGUGGAGGUGGUUCCUAATT‐3′ and 5'‐UUAGGAACCACCUCCACGCTT‐3′; TLR4 siRNA‐2, 5'‐CCTTTCCGGGACTTTCGCTTT‐3′ and 5′‐GUAGAATCCAGGTGGCAACA‐3′. SY5Y cells were transfected following a standard protocol.

### 2.7 RNA extraction and (SYBR green) real‐time PCR

Total cellular RNA was extracted from SY5Y cells using a TRI reagent. Total RNA was subjected to reverse transcription using reagents purchased from Promega (TaKaRa, Japan). GAPDH was used as the internal control for normalization. Relative expression was calculated as the fold change using the $2^{-\Delta \Delta C_{t}}$ method. The sequences of all primers used in this study are listed as follows (forward and reverse): TLR4, 5'‐AGGACTGGGTAAGGAATGAGC‐3′ and 5'‐ATCACCTTTCGGCTTTTATGG‐3′; Caspase‐1, 5'‐AATTTTCCGCAAGGTTCGATT‐3′ and 5'‐ACTCTTTCAGTGGTGGGCATCT‐3′; β‐actin, 5'‐CATGTACGTTGCTATCCAGGC‐3′ and 5'‐CTCCTTAATGTCACGCACGAT‐3′. The thermal cycling protocal was as follows: 95°C for 5 min, followed by 40 cycles at 95°C for 10 s and 60°C for 30 s.

### 2.8 Western blot analysis

Respiratory syncytial virus‐infected SY5Y cells were harvested at different time points for Western blot analysis, and uninfected SY5Y cells were defined as the negative control. 3 mL of precooled PBS (0.01 M, pH 7.2 ~ 7.3) was used to wash cells for 1 min before being discarded. The process was repeated twice. 1 mL of lysis buffer plus 10 μL of PMSF (100 mM) was then added to each cell sample along with 400 μL of pyrolysis buffer containing PMSF. The mixture was kept on ice for 30 min and centrifuged at 12,000 rpm for 5 min. The supernatant was collected and utilised for the detection of TLR3, RIG‐I and Hv1. Each sample was loaded with 40 micrograms of total protein, separated using 10% SDS–PAGE, and then transferred to 0.45 μm PVDF membranes (sc‐296,042, Santa Cruz Biotechnology, Inc., USA). The membranes were blocked with 5% nonfat dry milk in PBS containing 0.1% Tween 20 for 2 h, followed by the incubation with primary antibodies (1:500 dilution for TLR4, Caspase‐1, NLRP3 and LC3‐II. 1:1000 dilution for β‐actin) at 4°C overnight. The membranes were washed and further incubated with horseradish peroxidase‐conjugated anti‐rabbit or anti‐mouse secondary antibodies (1:10000, Santa Cruz Biotechnology) for 2 h. Enhanced chemiluminescence (Super Signal West Femto Substrate Kit, Thermo Scientific, USA) was employed to visualize immunoreactions in a Tanon 4500 automatic digital gel image analysis system (Tanon 4500, Shanghai, China). The relative intensity of each protein band was normalized to the corresponding β‐actin band and quantified using ImageJ software.

### 2.9 ELISA assay for IL‐1β, IL‐18 and TNF‐α

The concentrations of IL‐6, IL‐8 and TNF‐α in the culture medium from each group were determined by ELISA assay according to the manufacturer's instructions. Briefly, the culture supernatants were collected, and the concentrations of IL‐1β, IL‐18 and TNF‐α were determined using ELISA kits (Hermes Criterion Biotechnology, Vancouver, Canada).

### 2.10 Flow cytometric analysis

To investigate apoptosis in SY5Y neuronal cells, we conducted flow cytometry with Annexin V‐FITC and PI staining in accordance with the protocol. Infected cells were centrifuged at 2–8°C and 300 × *g* for 5 min, and the suspended cells were collected. The adherent cells were digested by pancreatic enzymes without EDTA and the digestion time was short enough to prevent false‐positives. Cells were washed twice with cold PBS and collected by centrifugation at 2–8°C and 300 × *g* for 5 min. The cells were then suspended in 400 μL of Annexin V reagent binding buffer (1 × 10^6^ cell/ml). The cells were incubated in a dark room for 15 min at 2–8°C after staining with 5 μL of Annexin V‐FITC and 10 μL of PI. Apoptosis was analysed by flow cytometry.

### 2.11 Statistical analysis

Data were presented as the mean ± SEM values. One‐way analysis of variance (ANOVA) followed by Fisher's post hoc test was applied to identify statistically significant differences between groups (SPSS 19.0; SPSS, Chicago, IL, USA). A *p*‐value < 0.05 was considered significant, and a *p*‐value < 0.01 was considered highly significant.

## 3 RESULTS

### 3.1 The immune response of neuronal cells was activated after RSV infection

The intercellular space was increased after SY5Y cells were treated with RSV. However, cellular synapses were absent or significantly diminished. Moreover, the number of cells was decreased compared with that of normal cells (Figure 2A). These results suggested that RSV had the ability to infect human neuronal cells and resulted in pathological changes.

**FIGURE 1:**
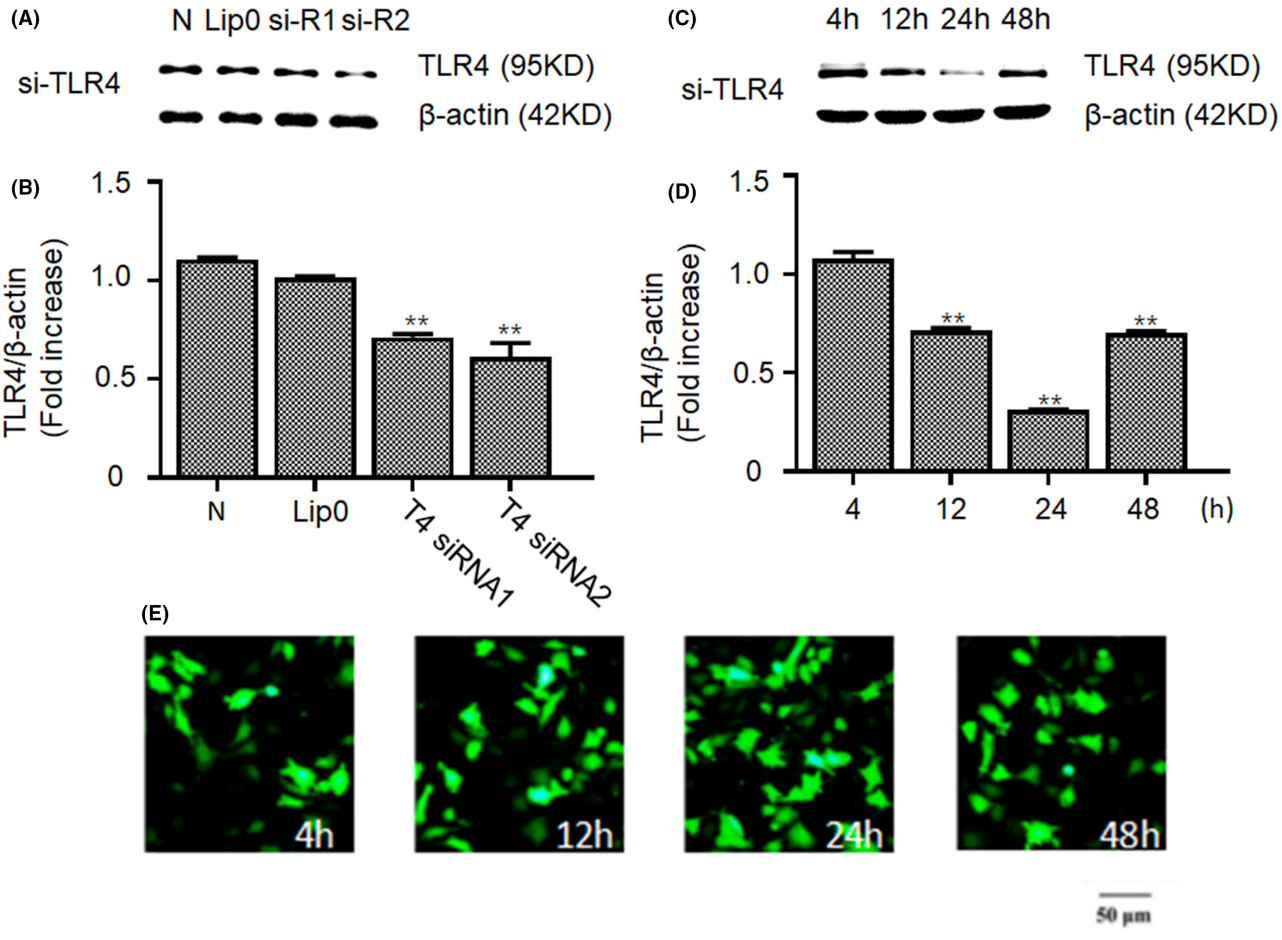
The effect of TLR4 siRNA transfection SY5Y cells. (A) Western blotting was used to detect TLR4 and β‐actin expression after TLR4 si‐R1 and si‐R2 transfection. N: normal control, Lip0: Lipofectamine 2000, si‐R1: TLR4 siRNA‐1, si‐R2: TLR4 siRNA‐2. (B) The relative expression of TLR4 was quantified by grayscale analysis. The protein levels in the indicated groups were presented relative to the β‐actin level (related to Figure 1A). (C) Western blotting was used to detect TLR4 and β‐actin expression at different time points after TLR4 siRNA transfection. (D) The relative expression of TLR4 was quantified by grayscale analysis. The protein levels at the indicated time points were presented relative to the β‐actin level (related to Figure 1C). (E) Representative IF staining of TLR4 siRNA at the indicated time points in SY5Y cells. Significance as determined by two‐tailed unpaired *t*‐tests is indicated in B and D. **p < 0.01.

The effects of the two TLR4 siRNA sequences, si‐R1 and si‐R2 sequences were evaluated using a Western blot analysis. Si‐R2 was selected for transfection experiments as it was better than si‐R1 (Figure 1A,B). TLR4 receptor expression was silenced by siRNA transfection, and the silencing efficiency at different time points was determined using an immunofluorescence assay (Figure 1E). Green fluorescence was observed at 4 h, 12 h, 24 h and 48 h after SY5Y cells were transfected with TLR4 siRNA, and the fluorescence peaked 24 h after transfection (Figure 1E). In addition, TLR4 protein expression was determined by Western blot analysis after isolating from siRNA‐silenced cells at various times after transfection. The results were consistent with the immunofluorescence results (Figure 1C,D).

**FIGURE 2:**
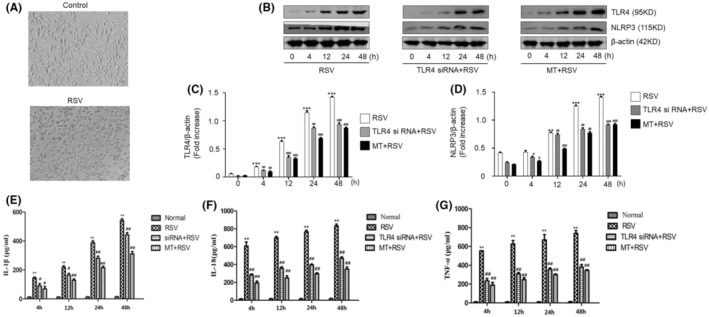
TLR4 mediates RSV infection and induces an inflammatory response in human neuronal cells. (A) Cytopathic effect in RSV‐infected SY5Y neuronal cells, compared with normal cells. (B) Western blotting was used to detect TLR4, NLRP3 and β‐actin expression at different time points after RSV infection, RSV infection combined with TLR4 siRNA transfection, and RSV infection combined with MT treatment.(C) The relative expression of TLR4 was quantified by grayscale analysis. The protein levels in the indicated groups were presented relative to the β‐actin level (related to Figure 3C). (D) The relative expression of NLRP3 was quantified by grayscale analysis. The protein levels in the indicated groups were presented relative to the β‐actin level (related to Figure 3C). (E–G) The concentrations of cytokines in SY5Y cells at different time points were detected by ELISA. IL‐1β, IL‐8 and TNF‐α were compared between the RSV group and the normal group. *p < 0.05, **p < 0.01; ^#^ *p* < 0.05, ^##^ *p* < 0.01.

The NLRP3 inflammasome is considered a key contributor to the development of neuroinflammation.^31^ In this study, we detected the expression of NLRP3 in neuronal cells after RSV infection. The results showed that NLRP3 was significantly upregulated after RSV infection (Figure 2B–D). Conversely, the expression of NLRP3 was decreased after TLR4 interference compared with that in the RSV infection group (Figure 2B–D). The ELISA results showed that the concentrations of the cytokines IL‐1β, IL‐18 and TNF‐α were significantly lower in the normal control group of SY5Y cells (Figure 2E–G). However, they were significantly increased in a time‐dependent manner after RSV infection (*p* < 0.01) and after TLR4 interference but were lower than those in the RSV infection group (Figure 2E–G).

These results demonstrated that the RSV could infect neuronal cells and induce immune responses. More importantly, TLR4 is critical for RSV infection‐induced immune responses in neuronal cells.

### 3.2 RSV infection induces apoptosis in neuronal cells

It has been reported that the cell death signalling pathways in dying cells regulate adaptive immunity.^32^ To determine whether immune responses associated with apoptosis occur concurrently in neuronal cells and are induced by RSV infection, the apoptosis marker caspase‐1 was evaluated. As expected, the results showed that at the 24 h and 48 h time points after RSV infection, caspase‐1 expression was significantly increased (Figure 4D,E). This finding was supported by the observation that the mRNA and protein expression levels of caspase‐1 were increased after RSV infection (Figure 4F). However, apoptosis rate was significantly lower at 24 h and 48 h when TLR4 was knocked down compared with that in the RSV‐infected group, with decreases of 4.77% at 24 h and 11% at 48 h (Figure 4A–C). The aforementioned data suggested that RSV infection induces apoptosis in SY5Y cells; however, transfection of TLR4 siRNA into neuronal SY5Y cells alleviates this phenomenon.

### 3.3 RSV infection induces autophagy in neuronal cells

A previous study demonstrated that smoke‐induced autophagy in epithelial cells appeared to promote airway inflammation.^33^ In the current paper, the expression of LC3‐II (an autophagy‐related marker) was also evaluated. The LC3‐II level was relatively low in the normal control group, but it was enhanced after infection with RSV and increased gradually in a time‐dependent manner (*p* < 0.01;Figure 5A,B). However, the LC3‐II level was significantly decreased after TLR4 interference compared with that in the RSV‐infected group (Figure 5A,B). Our findings indicated that RSV infection of SY5Y cells activates the receptors related to TLR4, initiates the host response and promotes autophagy. However, TLR4 interference obviously decreased the protein level of LC3‐II.

### 3.4 MT reverses the RSV infection‐induced cell response

**FIGURE 3:**
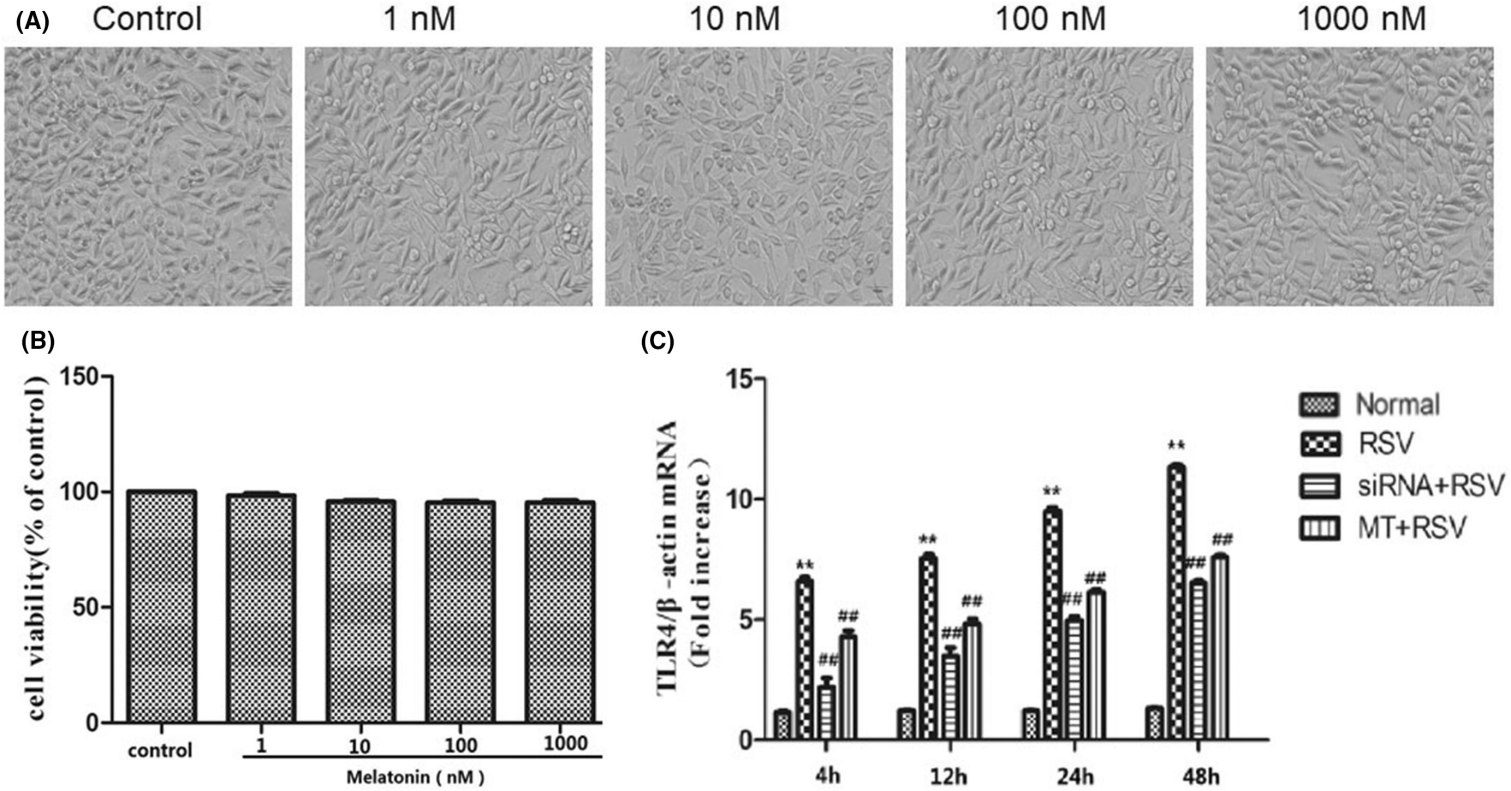
MT reverses RSV infection‐induced TLR4 upregulation in human neuronal cells. (A) After treatment with different concentrations (1 nmol/L, 10 nmol/L, 100 nmol/L and 1000 nmol/L) of MT, the morphology of SY5Y cells was compared with that of the normal cells. (B) SY5Y cells were treated with different concentrations of MT (1, 10, 100 or 1000 nM) for 24 h. The control cells were incubated with a medium for 24 h. Cell viability was measured by a CCK‐8 assay. (C) TLR4 mRNA expression in SY5Y cells in each group at different time points was evaluated by real‐time PCR. Significance as determined by two‐tailed unpaired *t*‐tests was indicated in B. *p < 0.05, **p < 0.01 compared with the normal group; ^#^ *p* < 0.05, ^##^ *p* < 0.01 compared with the RSV group.

It has been reported that an appropriate concentration of MT may help regulate different inflammatory signalling pathways in both tumour and normal tissues.^25^ Different MT concentrations were applied as treatments to investigate whether MT could protect neuronal cells against RSV infection. After treatment with different concentrations (1 nmol/L, 10 nmol/L, 100 nmol/L and 1000 nmol/L) of MT, SY5Y cells did not differ in morphology compared with normal cells (Figure 3A). In addition, the viability of cells treated with different concentrations of MT was evaluated by a CCK8 assay, and there were no significant differences compared with the control group, suggesting that the indicated concentration of MT had no toxicity in the SY5Y cells (Figure 3B). Finally, 1 nmol/L MT was selected as the suitable concentration.

First, we evaluated TLR4 expression after MT treatment in RSV‐infected SY5Y cells. The results indicated that the expression of TLR4 was significantly decreased at both the mRNA and protein levels compared with that in the RSV‐infected group (Figure 2D,E, Figure 3C). Second, we looked into whether MT played a role in reversing the RSV infection‐induced immune response. The results showed that MT significantly inhibited the RSV infection‐induced rises in IL‐1β, IL‐18 and TNF‐α (Figure 2A–C). Consistent with these findings, the expression of NLRP3 was also inhibited after MT treatment (Figure 2F,G).

**FIGURE 4:**
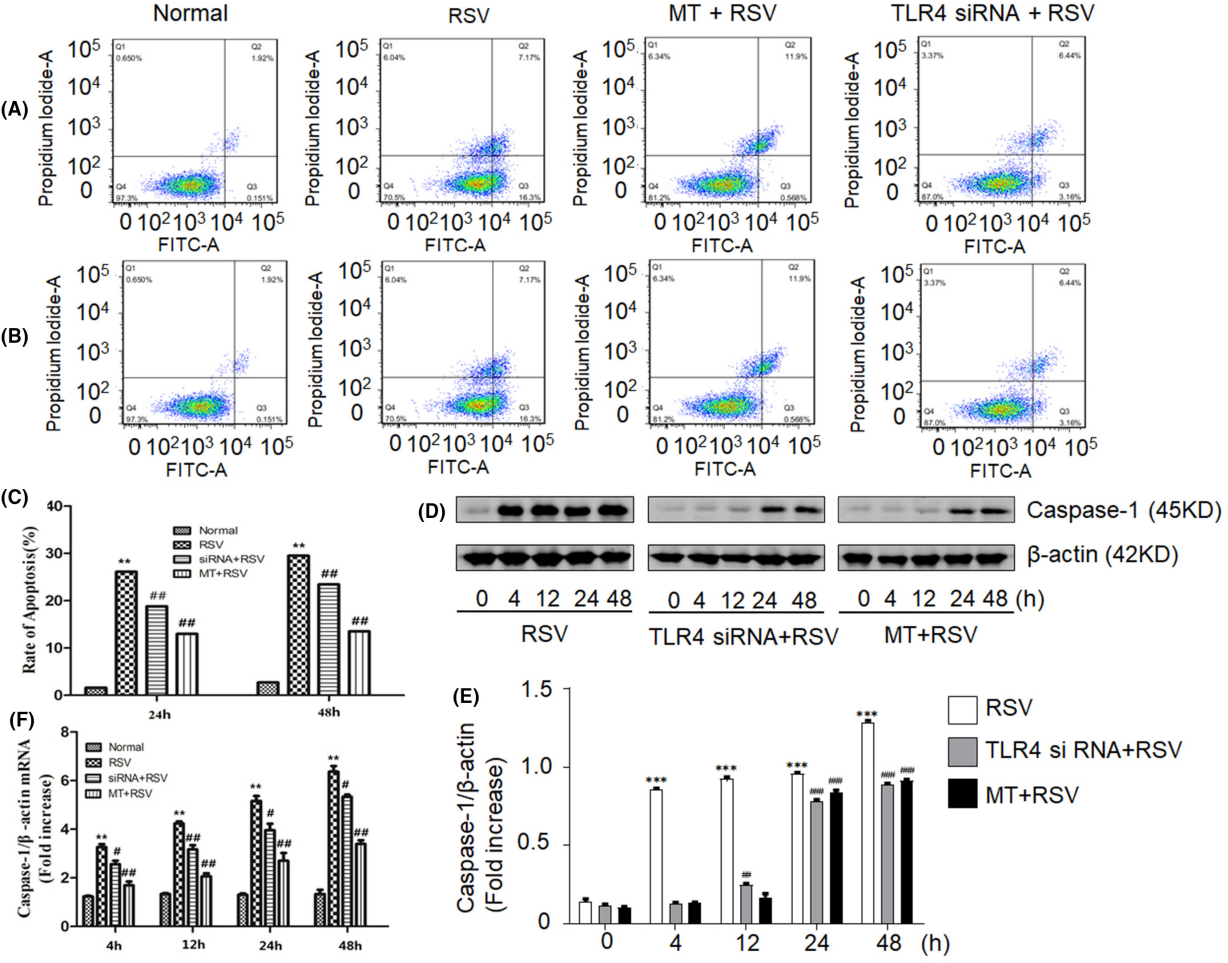
Human neuronal cells underwent apoptosis after RSV infection. (A) Apoptosis in SY5Y cells after RSV infection for 24 h. (B) Apoptosis in SY5Y cells after RSV infection for 48 h; Normal group (SY5Y cells without RSV infection); RSV group (SY5Y cells with RSV infection); TLR4 siRNA +RSV group (SY5Y cells with RSV infection and TLR4 siRNA transfection); MT + RSV group (SY5Y cells with RSV infection and MT treatment). (C) Quantification of the apoptosis rate in A and B. (D) Caspase‐1 protein expression in SY5Y cells at various time points after RSV infection in each group was evaluated by Western blot analysis (because they were from the same group of samples, the β‐actin shared with Figure 2B in the corresponding group). (E) The relative expression of Caspase‐1 was quantified by grayscale analysis. The protein levels in the indicated groups were presented relative to the β‐actin level (related to Figure 4B, D); (F) Caspase‐1 mRNA expression in SY5Y cells at various time points in each group was evaluated by real‐time PCR. *p < 0.05, **p < 0.01 compared with the normal group; ^#^ *p* < 0.05, ^##^ *p* < 0.01 compared with the RSV group.

**FIGURE 5:**
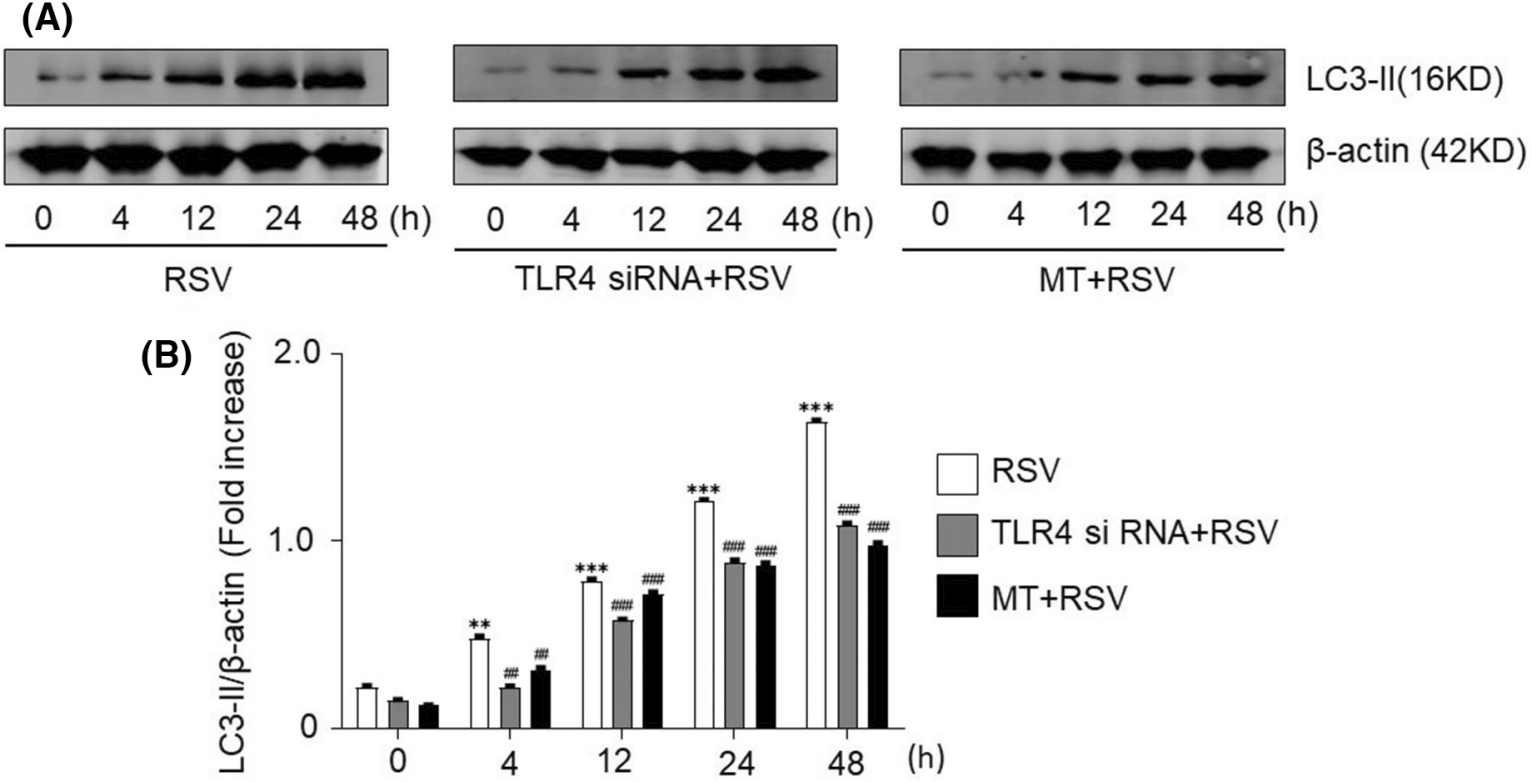
Human neuronal cells exhibited autophagy after RSV infection. (A) The LC3‐II protein levels in SY5Y cells at various time points after RSV infection in each group were evaluated by Western blot analysis (because they were from the same group of samples, the β‐actin shared with Figure 2B and 4D in the corresponding group). (B) The relative level of LC3‐II was quantified by grayscale analysis. The protein levels in the indicated groups were presented relative to the β‐actin level (related to Figure 5A). **p < 0.01, ***p < 0.001 compared with the normal group; ^##^ *p* < 0.01, ^###^ *p* < 0.001 compared with the RSV group.

**FIGURE 6:**
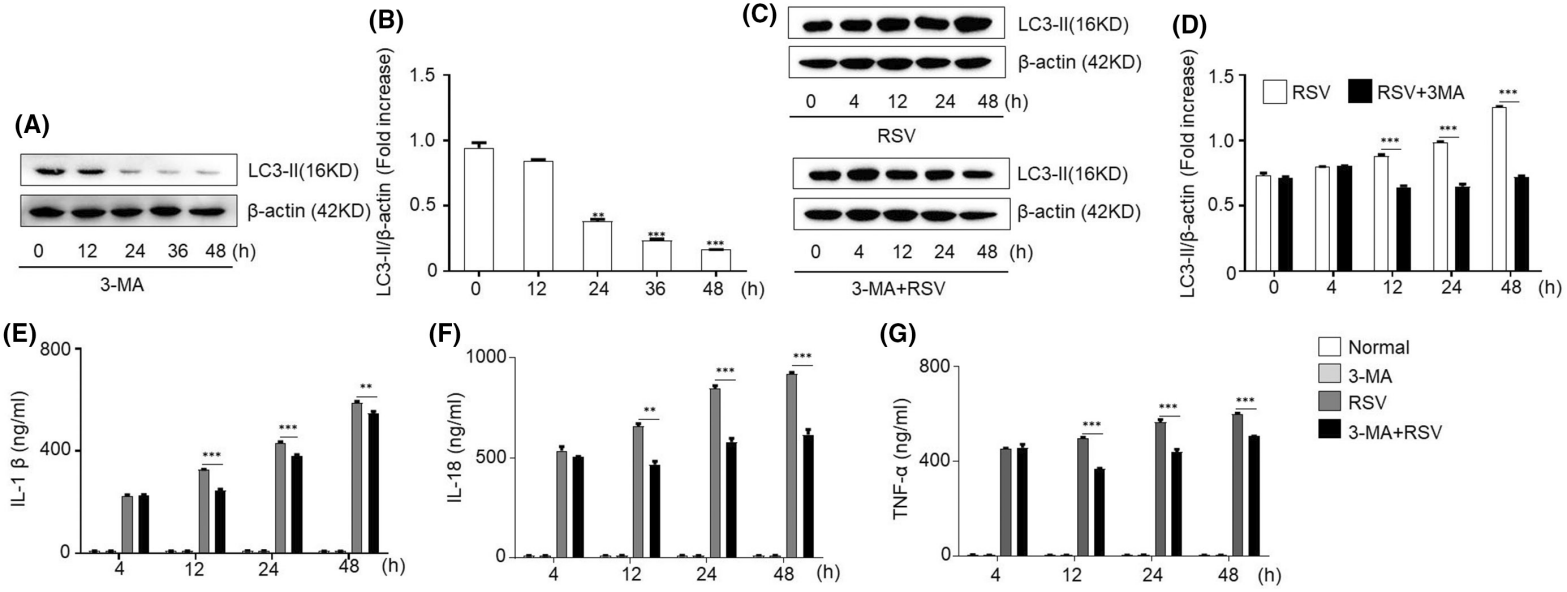
Autophagy is involved in RSV infection‐induced cytokine secretion. (A) Western blotting was used to detect LC3‐II after 3‐MA treatment at different time points. (B) The relative level of LC3‐II was quantified by grayscale analysis, The protein levels in the indicated groups were presented relative to the β‐actin level (related to Figure 6A). **p < 0.01 and **p < 0.001. (C) Western blot was used to detect LC3‐II protein at different time points after RSV infection and RSV infection combined with 3‐MA treatment. (D) The relative level of LC3‐II was quantified by grayscale analysis. The protein levels in the indicated groups were presented relative to the β‐actin level (related to Figure 6C). **p < 0.001. (E–G) The concentrations of cytokines in SY5Y cells at different time points, as measured by ELISA. IL‐1β, IL‐8 and TNF‐α were compared between the RSV group and the 3‐MA + RSV group: **p < 0.01 and ***p < 0.001.

Furthermore, we explored whether MT suppresses the immune response by preventing RSV infection‐induced apoptosis and autophagy. Strikingly, the results demonstrated that MT treatment significantly inhibited RSV infection‐induced apoptosis and autophagy (Figure 4A–E; Figure 5A,B). Furthermore, we investigated whether autophagy was involved in regulating inflammatory responses induced by RSV infection. To this end, we verified the inhibitory function of the cells by treating them with 3‐MA, an inhibitor of autophagy (Figure 6A,B). 3‐MA attenuated the autophagy induced by RSV infection in SY5Y cells (Figure 6C,D). Moreover, changes in the concentrations of the inflammatory factors IL‐1β, IL‐18 and TNF‐α were detected after 3‐MA treatment and RSV infection in SY5Y cells. The results illustrated that 3‐MA treatment attenuated the inflammatory response induced by RSV infection (Figure 6E–G).

In conclusion, the foregoing data demonstrated that TLR4 was critical for RSV infection‐induced immune responses in neuronal cells. RSV infection induced autophagy and apoptosis in neuronal cells. Autophagy was involved in regulating inflammatory responses induced by RSV infection. Furthermore, our findings demonstrated that MT suppresses TLR4‐mediated RSV infection in the CNS by inhibiting the autophagy‐mediated inflammatory response. This study proposed significant theoretical perspectives and broad application prospects for exploring the pathological mechanisms of RSV‐related encephalopathies, which may result in the development of a potential therapeutic strategy.

## 4 DISCUSSION

Studies have shown that the production of inflammatory factors induced by RSV infection is one of the causes of diseases in the CNS and pathological neurodegenerative diseases.^7^, ^34^, ^35^ The observation is consistent with our earlier research.^9^ In addition, MT may also regulate immunity and autophagy, exerting antioxidative and anti‐inflammatory effects according to some studies.^36^ However, whether MT plays a protective role in the RSV‐infected human nervous system through these effects is still unclear and is urgently needed to be elucidated.

RSV infection triggers intracellular signal transduction and induces the synthesis and release of a series of cytokines and inflammatory mediators to defend against viral infection, followed by inducing specific immune responses.^37^ Our study showed that RSV infection significantly upregulated the expression of TLR4 on neuronal cells, and the levels of downstream cytokines including IL‐1β, IL‐18 and TNF‐α were significantly increased. The results further confirmed that the inflammatory response was closely related to TLR4. Moreover, the levels of apoptosis and autophagy were significantly increased after activation of TLR4. Some studies have reported that influenza viruses result in encephalitis and encephalopathies, as the related neuronal cells release potentially cytotoxic substances such as TNF‐α, IL‐1β, oxygen radicals and nitric oxide, which also trigger apoptosis and autophagy in the infected cells.^38^ The findings suggested that inflammatory factors, such as IL‐1β, IL‐18 and TNF‐α, play an important role in the pathogenesis of encephalopathies. On the other hand, the occurrence and development of encephalopathies are closely related to TLR4‐induced activation of downstream pathways and autophagy in cells, indicating that TLR4 is crucial in RSV infection‐induced encephalopathies.

Autophagy is defined as a cellular process by which autologously damaged or aging organelles are degraded in lysosomes to maintain homeostasis, further allowing the metabolism of the cells and the renewal of some organelles.^39^, ^40^ We therefore investigated the autophagy marker LC3‐II, and the results of the experiments revealed that the level of LC3‐II was significantly increased in SY5Y cells after RSV infection. The levels of IL‐18, IL‐1β and TNF‐α decreased when autophagy was inhibited, suggesting that RSV may result in increased levels of nervous system inflammation through the TLR4‐induced autophagy pathway.

Recent studies have confirmed that MT deficiency significantly promotes the inflammatory response. In contrast, substantial MT may inhibit NLRP3 expression, which is also closely related to inflammation.^41^, ^42^ MT inhibit autophagic pathways as well according to additional studies.^43^ For example, neuronal cells are protected from death by MT in mouse models of cerebral ischemic injuries. MT may reduce the severity of autophagy by preventing the injury‐induced decrease in mTOR phosphorylation.^44^ In addition, MT inhibits the occurrence of autophagy in hepatocellular carcinoma cells and SK‐N‐SH cells.^45^, ^46^ The findings of our experiment were supported by the aforementioned studies, which showed that exogenous MT significantly decreased the protein levels of the inflammasome component NLRP3 and the autophagy marker LC3‐II in RSV‐infected SY5Y cells.

Melatonin significantly inhibited the expression of inflammatory factors, such as IL‐1β, IL‐18 and TNF‐α in a time‐dependent manner according to our research. Some researchers reported that MT treatment significantly decreased the level of phosphorylated NF‐κB and somewhat reduced the expression of NLRP3. These results indicated that NLRP3 may be involved in the regulatory effects of MT on cellular inflammation.^47^ To identify the signalling pathways that are involved in and mediate the regulation of the inflammasome by MT, the NF‐κB pathway was blocked indirectly by interfering with the expression of TLR4, which affected the activation of the NLRP3 inflammasome. In addition, the experiment revealed that MT provided a complete protective effect by suppressing the expression of TLR4 and activating the NLRP3 inflammasome in RSV‐infected human neuronal SY5Y cells. Other researchers discovered that MT plays a protective role in human tissues by eliminating free radicals, which interfere with the expression of NLRP3,^48^ offering new insight into the specific mechanism by which MT protects neurons.

Notably, there is a 10‐fold decrease in pineal melatonin release at night in the elderly, compared to adolescence,^49^ with similar dysregulated pineal melatonin in infants,^50^ suggesting that the increased severity of RSV lung infection in infants and elderly may also be linked to suppressed pineal melatonin production. This is supported by data showing melatonin to limit RSV infection in preclinical models.^51^ RSV infection occurs in astrocytes, brain endothelial cells and microglia as well as neurons.^52^ As astrocytes constitutively express and release melatonin,^53^ variations in the capacity of astrocytes to release melatonin may be a risk factor for CNS infection, as recently proposed for Alzheimer's disease.^54^ Our experiment clarified that exogenous MT provided a protective effect similar to the effect of blocking TLR4. However, the protective effect of MT was stronger than that of the monoblockade of TLR4. The effect may be achieved by inhibiting the expression of inflammatory factors produced by NLRP3 and the autophagy pathway, which would alleviate CNS inflammation. It may also be achieved by inhibiting the expression of inflammatory factors produced by other signalling pathways.

However, we acknowledge that there are some limitations in this study. For example, all experiments were conducted in vitro, and we did not establish a mouse model of RSV infection‐induced encephalopathy. Moreover, we did not evaluate the protective effect of MT on RSV encephalopathy in vivo, and this is a drawback of the experimental design of this study. In addition, by using specific inhibitors to suppress apoptosis progression, we are unable to explore the more specific mechanism by which MT regulates cellular apoptosis to further regulate inflammatory factors. That is, which pathway regulates inflammatory factors was not precisely determined. We hope that the present study will provide preliminary insight into the mechanisms of RSV infection‐induced encephalopathies and new ideas for the clinical treatment of RSV infection.

## 5 CONCLUSION

TLR4 was activated by RSV infection of human neuronal SY5Y cells, and the expression of NLRP3 and Caspase‐1 was promoted, thus activating downstream autophagy and further increasing the secretion of the inflammatory cytokines IL‐1β, IL‐18 and TNF‐α. Importantly, these findings revealed that the promotion of neuronal apoptosis and autophagy was significantly reversed after TLR4 silencing or exogenous MT treatment. Our results indicated that RSV promotes apoptosis and autophagy in central neuronal cells through TLR4. In addition, exogenous MT provides a good protective effect by inhibiting the activation of TLR4, suppressing the apoptosis programmes and inflammatory factor secretion caused by autophagy. Our research has significant implications for the future use of MT or TLR4 inhibitors in the prevention and treatment of virus‐induced neuroinflammation.

## AUTHOR CONTRIBUTIONS

**Yixuan Huang:** Investigation (equal); methodology (equal). **Chengcheng Jiang:** Data curation (equal); methodology (equal). **Xiaojie Liu:** Methodology (equal). **Wei Tang:** Writing – review and editing (equal). **Hongya Gui:** Investigation (equal); methodology (equal). **Tao Sun:** Methodology (equal). **Doudou Xu:** Data curation (equal). **Maozhang He:** Writing – original draft (equal). **Maozhen Han:** Writing – original draft (equal). **Huan Qiu:** Project administration (equal). **Mingwei Chen:** Project administration (equal). **Shenghai Huang:** Funding acquisition (lead); project administration (lead).

## FUNDING INFORMATION

This work was supported by Grants for Major Project of Natural Science Research of Anhui Education Department (No.KJ2019ZD23), The Fund of Excellent Talents in Colleges and Universities of Anhui Province (gxbjZD09), The Natural Science Foundation of China (No. 81974306, No. 81371797), The Scientific Research Foundation of Anhui Medical University (2020xkj018), Basic and Clinical Collaboration Enhancement Program Foundation of Anhui Medical University (2020xkjt023). Grants for Scientific Research of BSKY (XJ201922) from Anhui Medical University.

## CONFLICT OF INTEREST STATEMENT

The authors declare that they have no competing interests.

## CONSENT FOR PUBLICATION

All authors approved the publication of the article.

## Data Availability

All data generated or analysed during this study are included in this published article.
